# Network Based Approach in the Establishment of the Relationship between Type 2 Diabetes Mellitus and Its Complications at the Molecular Level Coupled with Molecular Docking Mechanism

**DOI:** 10.1155/2016/6068437

**Published:** 2016-09-06

**Authors:** Shailima Rampogu, Mary Rampogu Lemuel

**Affiliations:** ^1^Celesta Research Lab, Hyderabad, Telangana 500 076, India; ^2^West Thames College, London TW7 4HS, UK

## Abstract

Diabetes mellitus (DM) is one of the major metabolic disorders that is currently threatening the world. DM is seen associated with obesity and diabetic retinopathy (DR). In the present paper we tried to evaluate the relationship between the three aliments at the gene level and further performed the molecular docking to identify the common drug for all the three diseases. We have adopted several software programs such as Phenopedia, VennViewer, and CDOCKER to accomplish the objective. Our results revealed six genes that commonly associated and are involved in the signalling pathway. Furthermore, evaluation of common gene association from the selected set of genes projected the presence of SIRT1 in all the three aliments. Therefore, we targeted protein 4KXQ which was produced from the gene SIRT1 and challenged it with eight phytochemicals, adopting the CDOCKER. C1 compound has displayed highest -CDOCKER energy and -CDOCKER interaction energy of 43.6905 and 43.3953, respectively. Therefore, this compound is regarded as the most potential lead molecule.

## 1. Introduction

Globally, type 2 diabetes mellitus (T2DM) is one of the leading causes of death and it is estimated that over 70% of the population effected with T2DM are in the developing countries, China leading the world with 92.4 million [1, 2]. Once a patient is diagnosed with T2DM, lento develops the other diseases. It is generally noted that the T2DM patients are obese; however, the molecular association that exists between them is far from clear. Statistical data show an alarming figure, with 34% of the US adults being obese [3] and it may soon increase by 21% in another three decades [4]. It is well evidenced that obese men are more prone to develop T2DM than obese women at the ratio of 11.2 : 10. Nevertheless, it has to be noted that several patients with unrestrained weight again are not likely to develop T2DM, which warrants the need for understanding their relationship at the molecular level [3]. Another complication, which is seen associated with T2DM, is the diabetic retinopathy (DR) gradually causing visual impairment leading to blindness [5]. Recent reports evidently state the relationship between the BMI and DR [6]. Generally, BMI is considered as a determinant factor for obesity [7]. Wei et al. [8] also reported association between them at the genetic level. It is, hence, extremely essential to identify the relationship that exists between all the three diseases, majorly focusing on which disease is the initiator of the other two, and further to identify a common drug that could be a potential lead for all the diseases. To achieve this we have depended upon the system biology, which gives freedom in evaluating the biological system at the gene level. Additionally, this paper also makes an effort to identify the common genes and protein involved with the three aliments.

The objective of the present paper is to identify the genes representing the individual diseases and, further, the common genes involved and later to perform molecular docking to determine an effective drug molecule.

## 2. Materials and Methods

### 2.1. Identification of the Common Genes

To identify the common genes, we have employed Phenopedia [9], Public Healthy Genomics Knowledge Base (V1.0), to search for the genes responsible of the diseases and, consequently, the genes responsible for the three aliments were identified. Later, they were imported onto the pathway linkers [10] to understand the significant common genes associated with all the three diseases, which are precisely involved in the signalling pathways.

Furthermore, in order to understand the disease-gene relationship, we relied on VennViewer, provided with the Comparative Toxicogenomics Databases, which has an ability to develop the Venn diagrams pertaining to three genes, diseases, or chemicals. For the present investigation, we worked with three diseases.

### 2.2. Protein Ligand Docking

In order to identify the candidate drug molecules, it is very essential to perform the protein ligand docking, modelling technique used to predict the orientation, and the position of the ligand upon docking. For the present investigation, the CDOCKER, available on the Discovery Studio, was adopted. CDOCKER specifically employs the CHARMm-based molecular dynamics method and further generates conformation adopting the high temperature and is then forwarded onto the binding site for binding pose analysis.

### 2.3. Protein Selection and Preparation

The protein selection for the present investigation is one of the most crucial aspects. Since we aim at identifying the protein from the common gene, we relied upon 4KXQ, a protein produced from the gene SIRT1 with the resolution of 1.85 Å and is known as NAD-dependent protein deacetylase which envisages developing a common drug for all the three diseases.

The selected protein was prepared prior to the docking studies by correcting the chemistry of the missing hydrogens and the unfilled valence atoms. Thereafter, the protein was subjected to energy minimization by applying the CHARMm force field until a satisfactory gradient tolerance was obtained.

### 2.4. Ligand Preparation

A total of eight natural compounds were chosen to challenge against the protein target molecule. These compounds were drawn on Marvin Sketch and their corresponding 3D structures were generated on the DS. CHARMm force field was applied as a measure to minimize the ligand molecules. The importance of choosing the natural compounds is to further formulate and translate them into nutraceuticals, Table 1.

## 3. Results and Discussions

### 3.1. Identification of the Common Genes

Phenopedia was employed to identify the genes associated with obesity, diabetes mellitus, and diabetic retinopathy, respectively. A systematic search was conducted providing the disease names as a query. Consequently, SIRT1, MC4R, and VEGFA were determined for diabetes mellitus, type 2, obesity, and diabetic retinopathy, respectively.

**Figure 1:**
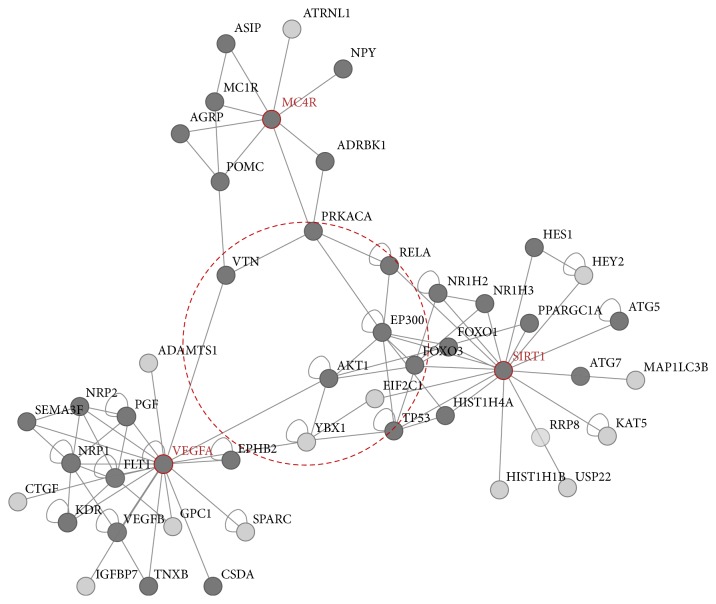
Red nodes indicate the query proteins and the proteins present in the dotted circles, VTN, PRKACA, RELA, EP300, AKT1, EIF2C1, are the common proteins associated with the query proteins. Dark grey nodes represent the signalling pathway members and the light grey nodes represent the nonsignalling pathway members.

Later, they were assessed for the common genes on* pathway linkers* that have an ability to link proteins to the signalling pathways. A total of 48 proteins were found to be associated; nevertheless, only six proteins were seen interacting with all the three genes. In addition, all the six proteins were involved in the signalling pathways and the nonsignalling proteins were ignored, Figure 1.

**Figure 2:**
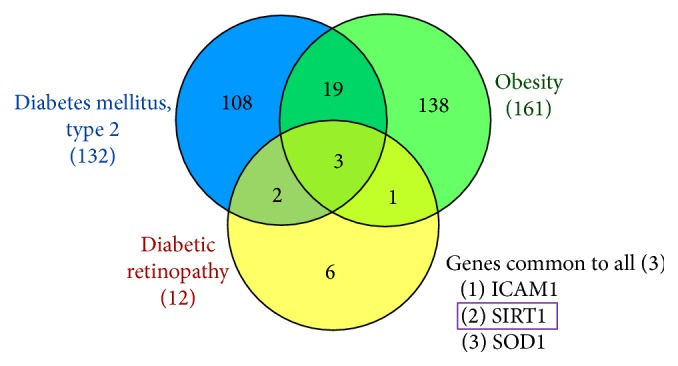
VennViewer diagram displaying the common genes associated with the three diseases.

Alternatively, we tried to evaluate the gene that is involved in all the three disorders and in this pursuit, we adopted the VennViewer [11] available on Comparative Toxicogenomics Database (CTD) facilitating the three genes of interest as inputs. Following which, the results were generated pronouncing SIRT1 to be involved with all the three diseases, Figure 2. Additionally, we have identified two other genes, ICAM1 and SOD1, that were seen involved with the three diseases.

### 3.2. Active Site Identification

**Figure 3:**
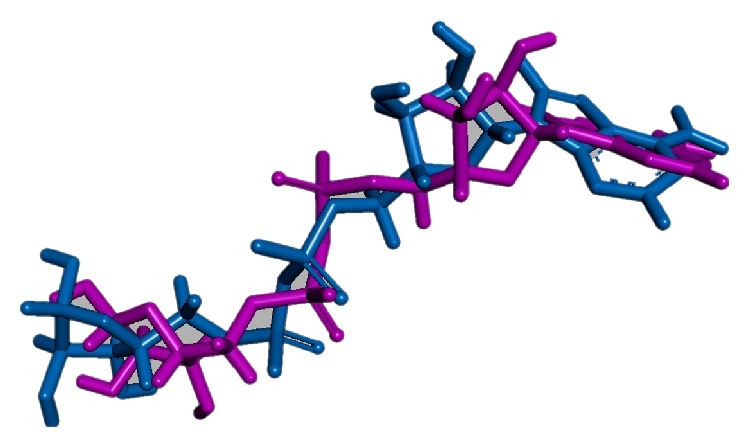
Docking of the cocrystal represented in magenta. Blue indicates the docked pose.

Active site was identified based upon the cocrystal and all the amino acids around the cocrystals were taken into consideration. Furthermore, the cocrystal docking was performed to ascertain the active site and an acceptable RMSD of 0.9 was obtained projecting that our docking parameters are valid ones, Figure 3.

### 3.3. Molecular Docking Mechanism

Molecular docking was performed adopting the CDOCKER, which depends on CHARMm-based force field. Subsequently, diverse poses are generated adopting the* random rigid body rotation* and simulate annealing. In order to initiate this mechanism, all the default parameters were considered allowing the generation of 10 poses for every ligand. The docking estimation was performed by the -CDOCKER energy, which was calculated, based upon the internal ligand strain energy and receptor-ligand interaction energy. Additionally, -CDOCKER interaction signifies the energy of the nonbonded interaction that exists between the protein and the ligand. In both the cases, it has to be noted that greater -CDOCKER energy and -CDOCKER interaction energy value implies greater favourable binding between the protein and the ligand.

**Figure 4:**
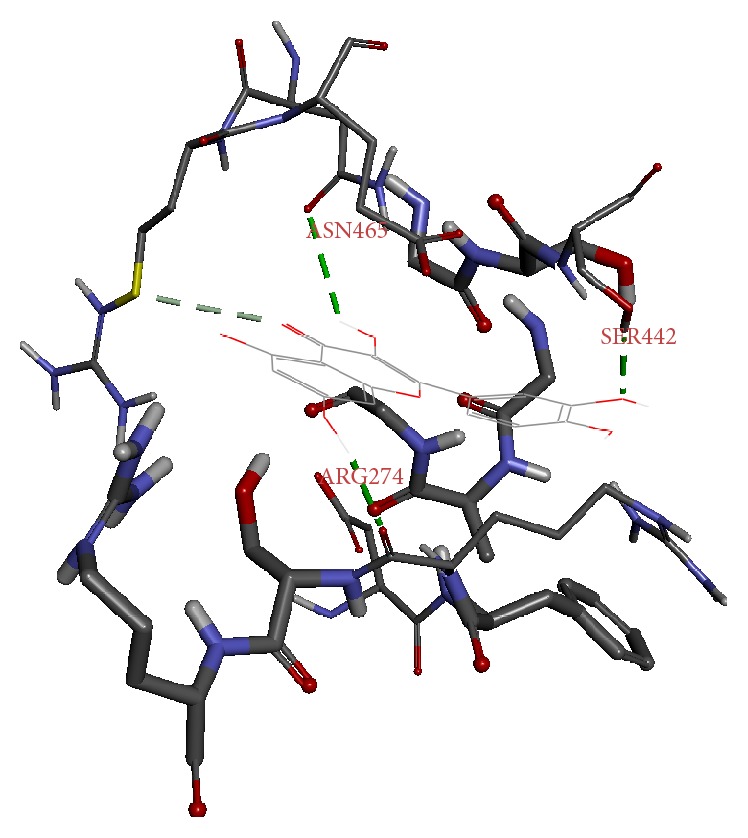
Protein ligand docking. Green dashed lines represent the hydrogen bond interactions.

**Figure 5:**
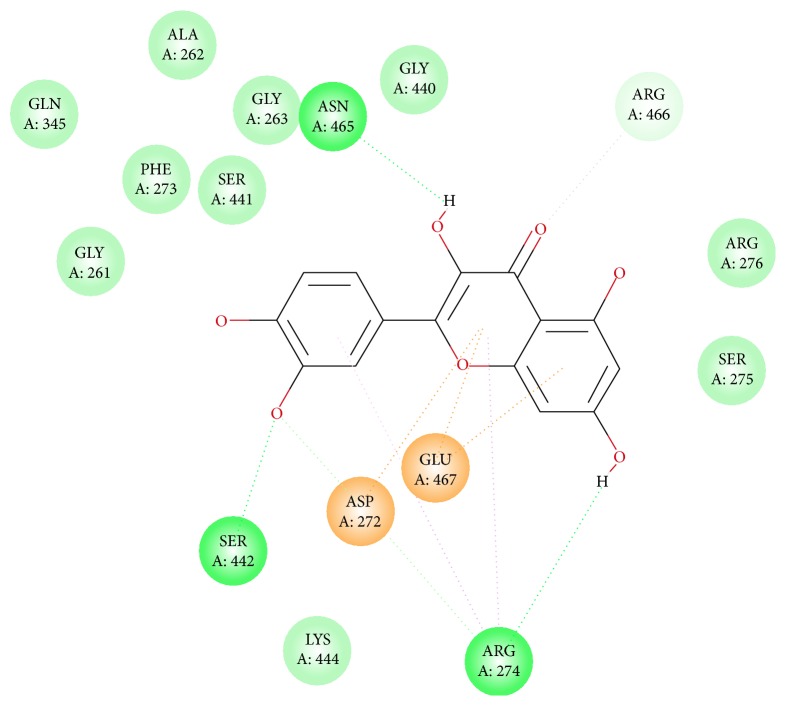
2D representation of the protein ligand interaction. Brown dotted lines indicate the pi-anion bonds, pink dotted lines denote the pi-alkyl bonds, and the light green dotted lines represent the van der Waals interactions.

As mentioned above, eight naturally available phytochemicals were challenged with the protein target. Seven ligands have displayed an efficient docking with the generation of 10 conformers each; however, one ligand failed to dock. The representative dock results are displayed in Table 2. Among the docked ligands, C1 displayed higher -CDOCKER energy and -CDOCKER interaction energy value-making itself the potential leading molecule for the three common diseases. Furthermore, the protein ligand complex was assessed for the hydrogen bond interaction followed by the binding mode analysis. Delineating on the interactions reveals that the residues, ASN465, SER442, and ARG274, have participated in the hydrogen bond formation, Figure 4, while the amino acid ARG466 participated by Vander Walls interaction and ASP272 and GLU467 interacted by the Pi-anion and ARG274 with Pi-alkyl bonds, respectively, Figure 5. Regardless of the binding energies projected by the ligands, they all obeyed the same pattern of binding mode as with the cocrystal.

## 4. Discussion

It has been a long subject of debate regarding the diabetes mellitus type 2 and its associated complication [12]. However, nothing concrete has yet been established. In the present paper, we have successfully evaluated the common genes associated with the three diseases. These findings could lead the researchers towards unfolding the mystery behind the diabetes complications. In the event of identifying the common genes associated among the three selected genes, our results determine SIRT1 to be the link gene, a gene which was chosen for diabetes mellitus. Therefore, it can be deduced that diabetes mellitus can influence the manifestation of obesity and diabetic retinopathy. Furthermore, our study centralizes on the identification of a common nutraceutical for all the three aliments. Accordingly, we have preferred the protein 4KXQ, a protein produced from the gene SIRT1. We have further evidenced the presence of SIRT1 in DM, obesity, and DR [13–15]. Following this, we challenged the selected protein with eight natural compounds or the phytochemicals. Natural compounds offer a host of applications such as low cost, wider availability, and low side effects which are a few to mention. Additionally, they can be supplemented through diet. Amongst all the ligand molecules, C1 emerged as the best ligand demonstrating a highest -CDOCKER interaction value of 46.3953 and its corresponding -CDOCKER energy of 43.6905, respectively.

In summary, our results signify being of greater scientific usefulness in finding the most prominent results in combating the diabetes complications.

## Figures and Tables

**Table 1:** Ligand name and structure.

Ligand name	Structure
C1	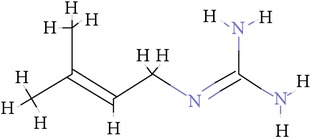

C2	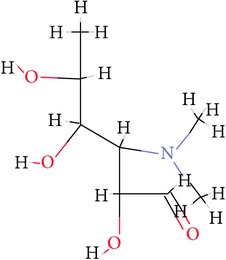

C3	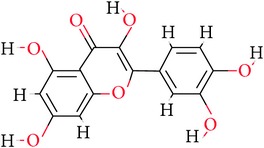

C4	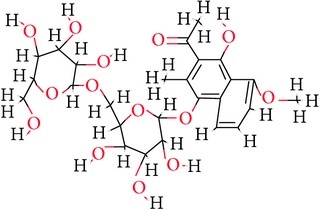

C5	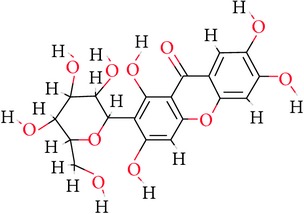

C6	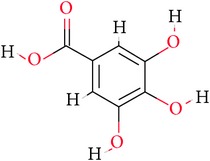

C7	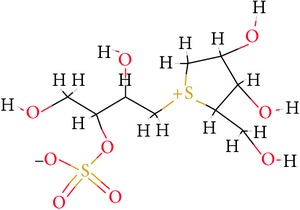

C8	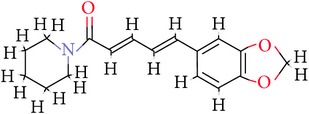

**Table 2:** CDOCKER scores.

Compound name	-CDOCKER energy	-CDOCKER interaction
C1	43.6905	46.3953
C2	43.594	46.2159
C3	43.4827	46.2751
C4	41.0953	43.8479
C5	39.5149	45.9837
C6	38.0443	41.3656
C7	37.9135	41.3597
C8	37.8987	41.12
C9	37.8849	41.0074
C10	36.7593	44.0819
C11	34.1467	30.8196
C12	34.0604	30.898
C13	33.9507	30.5325
C14	33.8314	30.5452
C15	33.7333	30.2313
C16	33.6998	30.4529
C17	33.6256	30.2404
C18	33.3807	29.9087
C19	33.3558	29.9264
C20	33.3249	29.8632
C21	16.1116	43.6807
C22	15.345	45.089
C23	13.6468	43.0185
C24	11.3323	33.3176
C25	11.117	29.8403
C26	10.8676	39.7459
C27	10.6943	36.1858
C28	6.76306	39.212
C29	6.74011	29.1499
C30	6.5533	28.9768
C31	6.37705	26.9035
C32	6.2874	27.1806
C33	6.10317	27.0072
C34	5.97667	26.6726
C35	5.92624	27.6206
C36	5.92438	26.961
C37	5.85956	26.796
C38	5.85111	26.9232
C39	5.82535	30.565
C40	5.72081	26.7008
C41	5.42032	42.1491
C42	5.2531	32.1618
C43	4.22422	40.4729
C44	3.15406	40.3832
C45	2.38138	36.5095
C46	2.31672	38.5722
C47	2.27477	36.9574
C48	1.9534	36.4364
C49	1.49815	36.2616
C50	1.18856	38.0515
C51	0.420181	22.2711
C52	0.183442	23.049
C53	0.0514005	48.6528
C54	−1.0173	26.8177
C55	−1.88654	45.5105
C56	−2.08119	47.3119
C57	−2.33309	48.7866
C58	−2.40939	41.621
C59	−3.58299	42.0705
C60	−4.44257	41.6539
C61	−4.72479	41.9058
C62	−4.92736	45.634
C63	−5.16567	44.7624
C64	−6.0176	39.6754
C65	−6.11149	39.7136
C66	−7.27294	42.9186
C67	−13.8456	32.8447
C68	−16.8805	32.324
C69	−19.8347	37.0598
C70	−19.8904	32.4273
